# Supplementary material for: Arbovirus Transmission in Australia from 2002 to 2017
Source: Biology (Basel). 2024 Jul 15;13(7):524. doi: 10.3390/biology13070524 (PMC11273437; doi:10.3390/biology13070524)
Supplement: Supplementary file 1 [file biology-13-00524-s001.zip › Revision Supl Mat/Supplementary Materials Doc S2 - Cluster analyses.pdf]

Arbovirus	Cluster number	SA3s included	Time frame	Observed cases	Expected cases	Obs/ Exp	Test statistic	Monte Carlo Rank	P-value
**BFV**	1	Pilbara, Kimberley, Goldfields, Mid West, Gascoyne, Outback - North and East, Wheat Belt - North, Mundaring, Wanneroo, Swan, Joondalup, Kalamunda, Bayswater - Bassendean, Stirling, Perth City, Cottesloe - Claremont, South Perth, Canning, Gosnells, Melville, Fremantle, Armadale, Cockburn, Kwinana, Rockingham, Serpentine - Jarrahdale, Mandurah, Alice Springs, Esperance, Bunbury, Wheat Belt - South, Daly - Tiwi - West Arnhem, Albany, Manjimup, Belmont - Victoria Park, Augusta - Margaret River - Busselton, Darwin Suburbs, Palmerston, Litchfield, Darwin City	2013/1/1 to 2013/12/31	1355	622.66	2.18	333.23	1/1000	< 1e-17

	2	Grampians, Maryborough - Pyrenees, Barwon - West, Ballarat, Creswick - Daylesford - Ballan, Glenelg - Southern Grampians, Surf Coast - Bellarine Peninsula, Warrnambool - Otway Ranges, Limestone Coast, Bendigo, Heathcote - Castlemaine - Kyneton, Geelong, Wyndham, Campaspe, Loddon - Elmore, Sunbury, Melton - Bacchus Marsh, Tullamarine - Broadmeadows, Hobsons Bay, Brimbank, Keilor, Mornington Peninsula, Maribyrnong, Essendon, Moreland - North, Brunswick - Coburg, Melbourne City, Darebin - South, Stonnington - West, Yarra, Boroondara, Darebin - North, Banyule, Whittlesea - Wallan, Stonnington - East, Glen Eira, Bayside, Monash, Kingston, Whitehorse - West, Mildura, Whitehorse - East, Manningham - West, Manningham - East, Maroondah, Dandenong, Frankston, Knox, Casey - South, Casey - North, Upper Murray exc. Albury, Shepparton, Nillumbik - Kinglake, Cardinia, Port Phillip, Upper Goulburn Valley, Moira, Baw Baw, Murray River - Swan Hill, Lower Murray, Macedon Ranges, Wellington, Wangaratta - Benalla, Latrobe Valley, Fleurieu - Kangaroo Island, Gippsland - South West, Wodonga - Alpine, Albury, Adelaide Hills, Murray and Mallee, Onkaparinga, Gippsland - East, Mitcham, Unley, Campbelltown (SA), Burnside, Marion, Norwood - Payneham - St Peters, Adelaide City, Tea Tree Gully, Holdfast Bay, Prospect - Walkerville, Port Adelaide - East, West Torrens, Playford, Port Adelaide - West, Charles Sturt, Salisbury, Barossa, Gawler - Two Wells, Lower North, Tumut - Tumbarumba, Yorke Peninsula, Burnie - Ulverstone, Wagga Wagga, Lower Hunter, Inverell - Tenterfield, West Coast, Broken Hill	2013/1/1 to 2013/12/31	567	1304.28	0.43	277.19	1/1000	< 1e-17

		and Far West, Griffith - Murrumbidgee (West), Snowy Mountains, Devonport, Meander Valley - West Tamar, Launceston, Bourke - Cobar - Coonamble, Lachlan Valley, Mid North, South Coast, North East, Eyre Peninsula and South West, Queanbeyan, Central Highlands (Tas.), Brighton, Hobart - North West, Hobart - North East, Goulburn - Yass, Hobart Inner, Sorell - Dodges Ferry, South East Coast, Huon - Bruny Island, Hobart - South and West, Kiama - Shellharbour, Jervis Bay, Shoalhaven, Blue Mountains - South, Illawarra Catchment Reserve, Wollondilly, Southern Highlands, Wollongong, Dapto - Port Kembla, Campbelltown (NSW), Lithgow - Mudgee, Camden, Penrith, Liverpool, Blue Mountains, Bathurst, Bringelly - Green Valley, Richmond - Windsor, St Marys, Auburn, Fairfield, Cronulla - Miranda - Caringbah, Sutherland - Menai - Heathcote, Mount Druitt, Merrylands - Guildford, Blacktown - North, Rouse Hill - McGraths Hill, Bankstown, Hurstville, Kogarah - Rockdale, Blacktown, Canterbury, Parramatta, Canada Bay, Baulkham Hills, Lake Macquarie - East, Pennant Hills - Epping, Marrickville - Sydenham - Petersham, Leichhardt, Carlingford, Botany, Eastern Suburbs - South, Ryde - Hunters Hill, Dural - Wisemans Ferry,Chatswood - Lane Cove, Sydney Inner City, Hornsby, Eastern Suburbs - North, Ku-ring-gai, Hawkesbury, North Sydney - Mosman, Warringah, Pittwater, Gosford, Tamworth - Gunnedah, Upper Hunter, Lake Macquarie - West, Wyong, Manly, Dubbo, Newcastle, Strathfield - Burwood - Ashfield, Maitland, Great Lakes, Taree - Gloucester, Moree - Narrabri, Port Macquarie, Armidale, Kempsey -							

		Nambucca, Coffs Harbour, Clarence Valley, Richmond Valley - Hinterland, Granite Belt, Orange, Outback - South, Port Stephens, Darling Downs (West) - Maranoa, Richmond Valley - Coastal, Beaudesert, Sherwood - Indooroopilly, Gold Coast Hinterland							

	3	Bowen Basin - North, Charters Towers - Ayr - Ingham, Whitsunday, Innisfail - Cassowary Coast, Outback - North, Townsville, Rockhampton, Central Highlands (Qld), Tablelands (East) - Kuranda, Cairns - South, Yarra Ranges, Cairns - North, Port Douglas - Daintree	2016/1/1 to 2016/12/31	114	51.89	**2.2**	27.71	1/1000	< 76e-17
**DENV**	1	Innisfail - Cassowary Coast	2011/1/1 to 2011/12/31	51	4.91	**10.39**	73.67	1/1000	< 1e-17
	2	Port Douglas - Daintree	2013/1/1 to 2013/12/31	30	4.76	**6.31**	30.11	1/1000	< 47e-14
	3	Tablelands (East) - Kuranda, Yarra Ranges, Cairns - North	2009/1/1 to 2009/12/31	121	63.49	1.91	21.12	1/1000	< 16e-9
**RRV**	1	Limestone Coast, Glenelg - Southern Grampians, Grampians, Mildura, Fleurieu - Kangaroo Island, Maryborough - Pyrenees, Barwon - West, Onkaparinga, Adelaide Hills, Murray and Mallee, Ballarat, Mitcham, Unley, Campbelltown (SA), Burnside, Marion, Norwood - Payneham - St Peters, Adelaide City, Holdfast Bay, Tea Tree Gully, Prospect - Walkerville, West Torrens, Port Adelaide - East, Charles Sturt, Port Adelaide - West, Upper Murray exc. Albury, Salisbury, Playford, Bendigo, Heathcote - Castlemaine - Kyneton, Creswick - Daylesford - Ballan, Murray River - Swan Hill, Surf Coast - Bellarine Peninsula, Barossa, Campaspe, Loddon - Elmore, Gawler - Two Wells, Warrnambool - Otway Ranges, Yorke Peninsula, Geelong, Wyndham, Sunbury, Lower North	2011/1/1 to 2011/12/31	2037	456.78	**4.46**	1481.93	1/1000	< 1e-17

	2	Browns Plains, Jimboomba, Rocklea - Acacia Ridge, Springwood - Kingston, Mt Gravatt, Sunnybank, Loganlea - Carbrook, Nathan, Southport, Holland Park - Yeronga, Centenary, Forest Lake - Oxley, Beenleigh, Ormeau - Oxenford, Ipswich Inner, Springfield - Redbank, Brisbane Inner, Brisbane Inner - West, Kenmore - Brookfield - Moggill, Carindale, Capalaba, Brisbane Inner - North, Brisbane Inner - East, Far North, The Gap - Enoggera, Chermside, Hills District, Cleveland - Stradbroke, Nundah, Ipswich Hinterland, Wynnum - Manly, Sandgate, Bald Hills - Everton Park, Beaudesert, Nerang, Strathpine, Gold Coast - North, North Lakes, Gold Coast Hinterland, Redcliffe, Surfers Paradise, Robina, Broadbeach - Burleigh, Toowoomba, Narangba - Burpengary, Mudgeeraba - Tallebudgera, Coolangatta, Sherwood - Indooroopilly, Caboolture, Bribie - Beachmere, Tweed Valley	2015/1/1 to 2015/12/31	4202	2050.09	2.05	895.63	1/1000	< 1e-17
	3	Pilbara, Kimberley, Goldfields, Mid-West, Gascoyne, Outback - North and East, Wheat Belt - North, Mundaring, Wanneroo, Swan, Joondalup, Kalamunda, Bayswater - Bassendean, Stirling, Perth City, Cottesloe - Claremont, South Perth, Canning, Gosnells, Melville, Fremantle, Armadale, Cockburn, Kwinana, Rockingham, Serpentine - Jarrahdale, Mandurah, Alice Springs, Esperance, Bunbury, Wheat Belt - South, Daly - Tiwi - West Arnhem, Albany, Manjimup, Belmont - Victoria Park, Augusta - Margaret River - Busselton, Darwin Suburbs, Palmerston, Litchfield	2012/1/1 to 2014/12/31	4970	2993.21	1.66	570.58	1/1000	< 1e-17
**WNV**	1	Townsville, Innisfail - Cassowary Coast, Outback - North, Charters Towers - Ayr - Ingham, Bowen Basin - North, Tablelands (East) - Kuranda,	2010/1/1 to 2017/12/31	0	3.08	0	**3.51**	13/1000	0.013

		Cairns - South, Yarra Ranges, Cairns - North, Whitsunday, Port Douglas - Daintree, Burnett, Central Highlands (Qld), Rockhampton, Outback - South, Gladstone - Biloela, Bundaberg, Darling Downs (West) - Maranoa, Hervey Bay, Caboolture Hinterland, Maryborough, Mackay, Noosa, Katherine, Gympie - Cooloola, Nambour - Pomona, Sunshine Coast Hinterland, Maroochy, Buderim, Caloundra, Darling Downs - East, Caboolture, Narangba - Burpengary, Toowoomba, Bribie - Beachmere, Sherwood - Indooroopilly, Granite Belt, Chermside, Kenmore - Brookfield - Moggill, Brisbane Inner - West Brisbane Inner - North, Brisbane Inner, Brisbane Inner - East, Far North							
	2	Mandurah, Serpentine - Jarrahdale, Rockingham, Kwinana, Cockburn, Armadale, Melville, Canning, Gosnells, Fremantle, South Perth, Perth City, Cottesloe - Claremont, Bayswater - Bassendean, Kalamunda, Stirling, Swan, Joondalup, Bunbury, Mundaring, Wanneroo, Belmont - Victoria Park, Augusta - Margaret River - Busselton, Wheat Belt - North, Manjimup, Wheat Belt - South, Albany, Esperance, Mid West, Gascoyne, Goldfields, Pilbara, Kimberley, Outback - North and East, Eyre Peninsula and South West, Alice Springs, Yorke Peninsula, Holdfast Bay, Charles Sturt, West Torrens, Marion, Port Adelaide - West, Adelaide City, Prospect - Walkerville, Burnside, Mitcham, Norwood - Payneham - St Peters, Unley, Salisbury, Port Adelaide - East, Campbelltown (SA), Playford, Tea Tree Gully, Onkaparinga, Gawler - Two Wells, Barossa, Mid North, Adelaide Hills, Murray and Mallee, Lower North, Fleurieu - Kangaroo Island, Limestone	2003/1/1 to 2010/12/31	0	2.77	0	3.11	48/1000	0.048

		Coast, Glenelg - Southern Grampians, Mildura, Barkly, Grampians							
**MVEV**	1	Katherine, Barkly	2015/1/1 to 2015/12/31	2	0.2	**10**	2.86	63/95	0.53
	2	Esperance, Albany, Wheat Belt - South, Manjimup, Bunbury, Belmont - Victoria Park, Serpentine - Jarrahdale, Mandurah, Armadale, Mundaring, Kwinana, Cockburn, Kalamunda, Rockingham, Canning, Gosnells, Melville, South Perth, Bayswater - Bassendean, Augusta - Margaret River - Busselton, Swan, Perth City, Fremantle, Stirling, Cottesloe - Claremont, Joondalup, Wanneroo, Wheat Belt - North, Mid West, Gascoyne, Goldfields, Pilbara, Eyre Peninsula and South West, Yorke Peninsula, Kimberley, Holdfast Bay, West Torrens, Charles Sturt, Marion, Port Adelaide - West, Adelaide City, Mitcham, Prospect - Walkerville, Burnside, Norwood - Payneham - St Peters, Unley, Salisbury, Port Adelaide - East, Campbelltown (SA), Outback - North and East, Onkaparinga, Tea Tree Gully, Playford, Gawler - Two Wells, Barossa, Adelaide Hills, Mid North, Murray and Mallee	2002/1/1 to 2005/12/31	0	2.5	0	2.61	70/95	0.73

	3	Outback - South, Central Highlands (Qld), Rockhampton, Innisfail - Cassowary Coast, Outback - North, Charters Towers - Ayr - Ingham, Darling Downs (West) - Maranoa, Whitsunday, Townsville, Bowen Basin - North, Gladstone - Biloela, Tablelands (East) - Kuranda, Bundaberg, Moree - Narrabri, Mackay, Granite Belt, Darling Downs - East, Caboolture Hinterland, Maryborough, Sherwood - Indooroopilly, Hervey Bay, Toowoomba, Sunshine Coast Hinterland, Broken Hill and Far West, Nambour - Pomona, Gympie - Cooloola, Ipswich Hinterland, Noosa, Yarra Ranges, Caboolture, Maroochy, Cairns - South, Narangba - Burpengary, Cairns - North, Ipswich Inner, Springfield - Redbank, Buderim, Strathpine, Kenmore - Brookfield - Moggill, Caloundra, Hills District, Dubbo	2005/1/1 to 2008/12/31	2	0.33	**6**	1.96	91/95	0.96
	4	Daly - Tiwi - West Arnhem, Darwin Suburbs, Palmerston, Litchfield, Darwin City, East Arnhem	2011/1/1 to 2015/12/31	0	1.8	0	1.86	93/95	0.98

Obs/Exp = is the estimated risk within the cluster divided by the estimated risk for the study region.

*P-value < 0.05.

Most likely cluster bolded.
