# Supplementary material for: Arbovirus Transmission in Australia from 2002 to 2017
Source: Biology (Basel). 2024 Jul 15;13(7):524. doi: 10.3390/biology13070524 (PMC11273437; doi:10.3390/biology13070524)
Supplement: Supplementary file 1 [file biology-13-00524-s001.zip › Revision Supl Mat/Supplementary Materials Figure S1 - Arboviruses distribution.pdf]

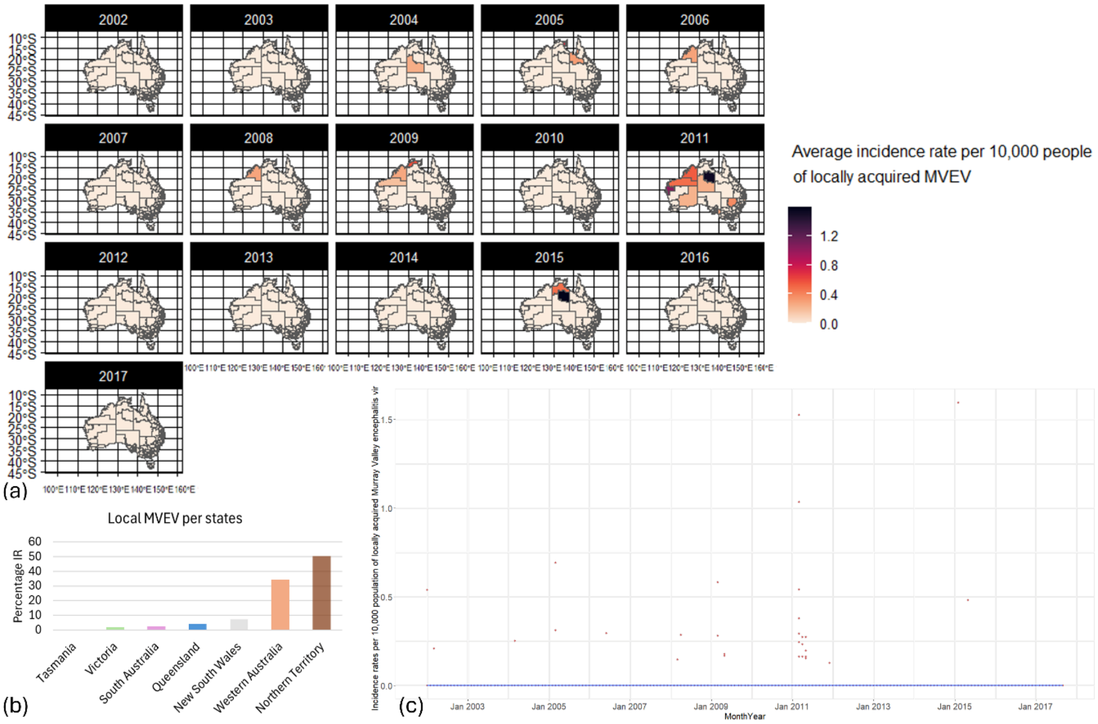

Murray Valley encephalitis virus distribution from 2002 to 2017 in Australia. (**a**) Maps of the distribution of MVEV cases per year through SA3s; (**b**) Top three states with the highest percentage of MVEV local incidence rate; (**c**) Distribution of MVEV incidence rate per 10,000 population through Australia; red dots: Incidence rate per 10,000 population per time and space; blue line: smoothed line to the plot. By default, geom_smooth() uses a Loess smoothing method for small datasets and a generalized additive model for larger datasets

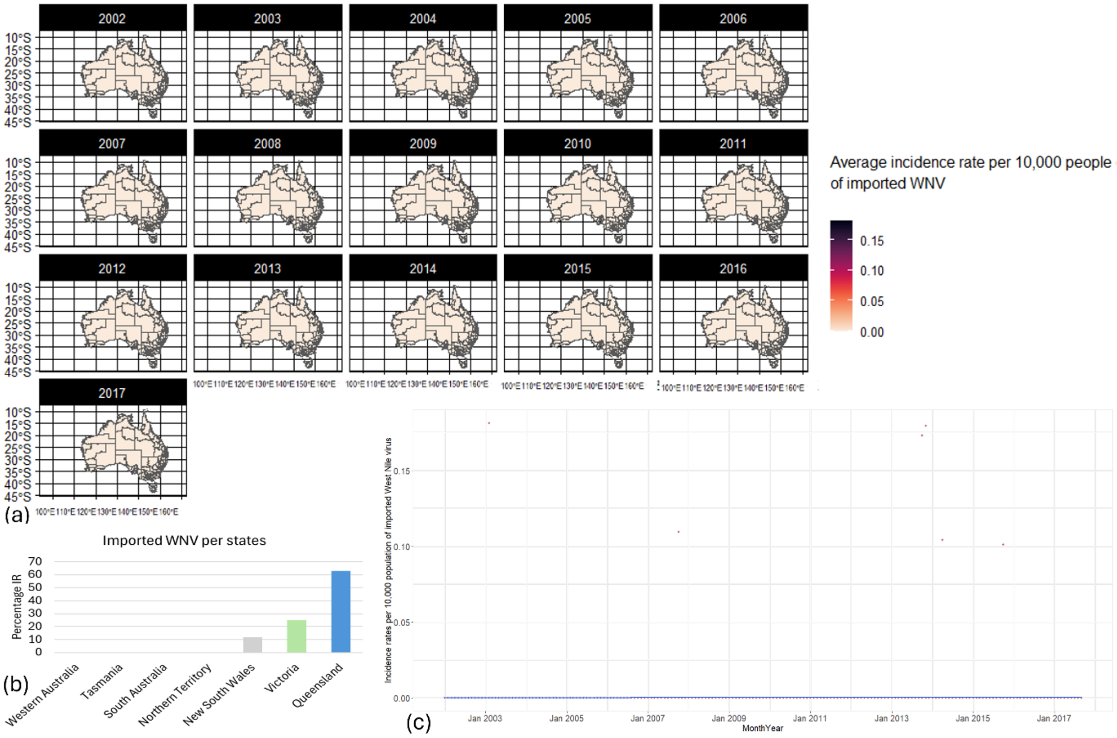

Imported West Nile virus distribution from 2002 to 2017 in Australia. (**a**) Maps of the distribution of imported WNV cases per year through SA3s; (**b**) Top three states with the highest percentage of imported WNV local incidence rate; (**c**) Distribution of imported WNV incidence rate per 10,000 population through Australia; red dots: Incidence rate per 10,000 population per time and space; blue line: smoothed line to the plot. By default, geom_smooth() uses a Loess smoothing method for small datasets and a generalized additive model for larger datasets

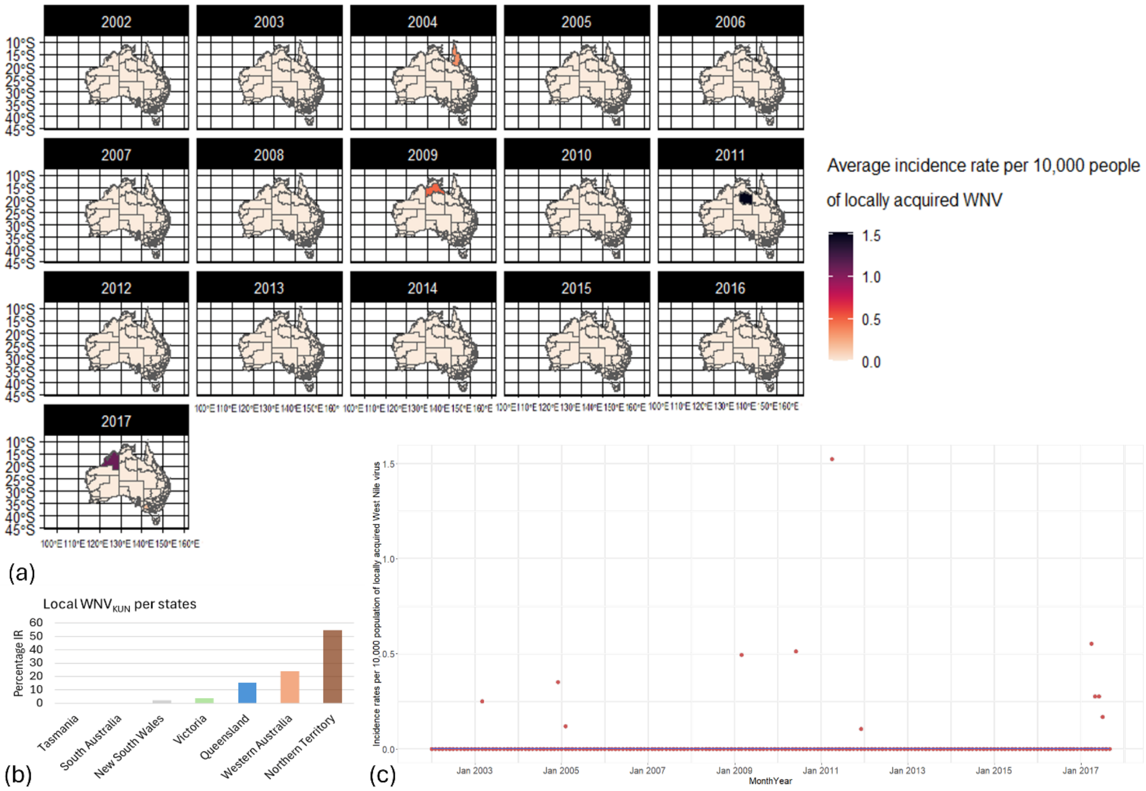

Locally acquired West Nile virus/Kunjin strain distribution from 2002 to 2017 in Australia. (**a**) Maps of the distribution of locally acquired WNV_{KUN} cases per year through SA3s; (**b**) Top three states with the highest percentage of locally acquired WNV_{KUN} local incidence rate; (**c**) Distribution of locally acquired WNV_{KUN} incidence rate per 10,000 population through Australia; red dots: Incidence rate per 10,000 population per time and space; blue line: smoothed line to the plot. By default, geom_smooth() uses a Loess smoothing method for small datasets and a generalized additive model for larger datasets

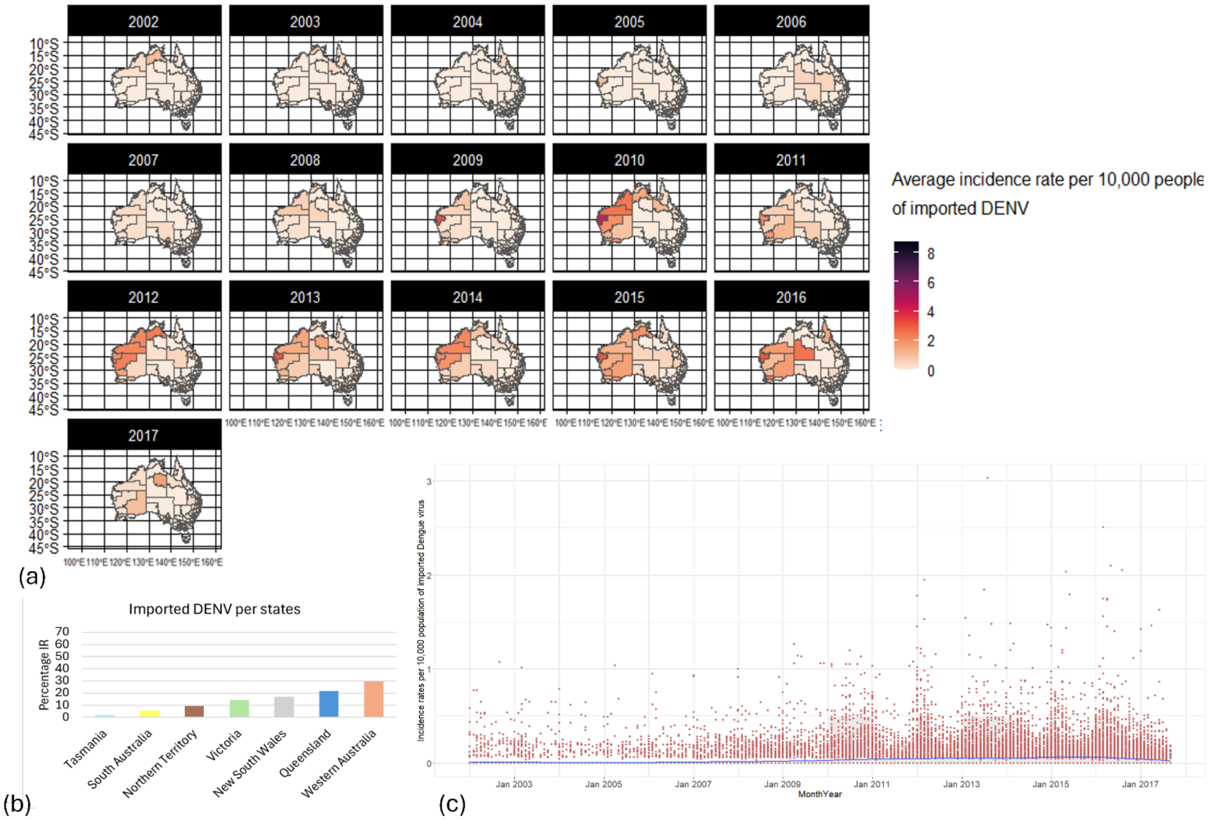

Imported dengue virus distribution from 2002 to 2017 in Australia. (**a**) Maps of the distribution of Imported DENV cases per year through SA3s; (**b**) Top three states with the highest percentage of imported DENV local incidence rate; (**c**) Distribution of imported DENV incidence rate per 10,000 population through Australia; red dots: Incidence rate per 10,000 population per time and space; blue line: smoothed line to the plot. By default, geom_smooth() uses a Loess smoothing method for small datasets and a generalized additive model for larger datasets

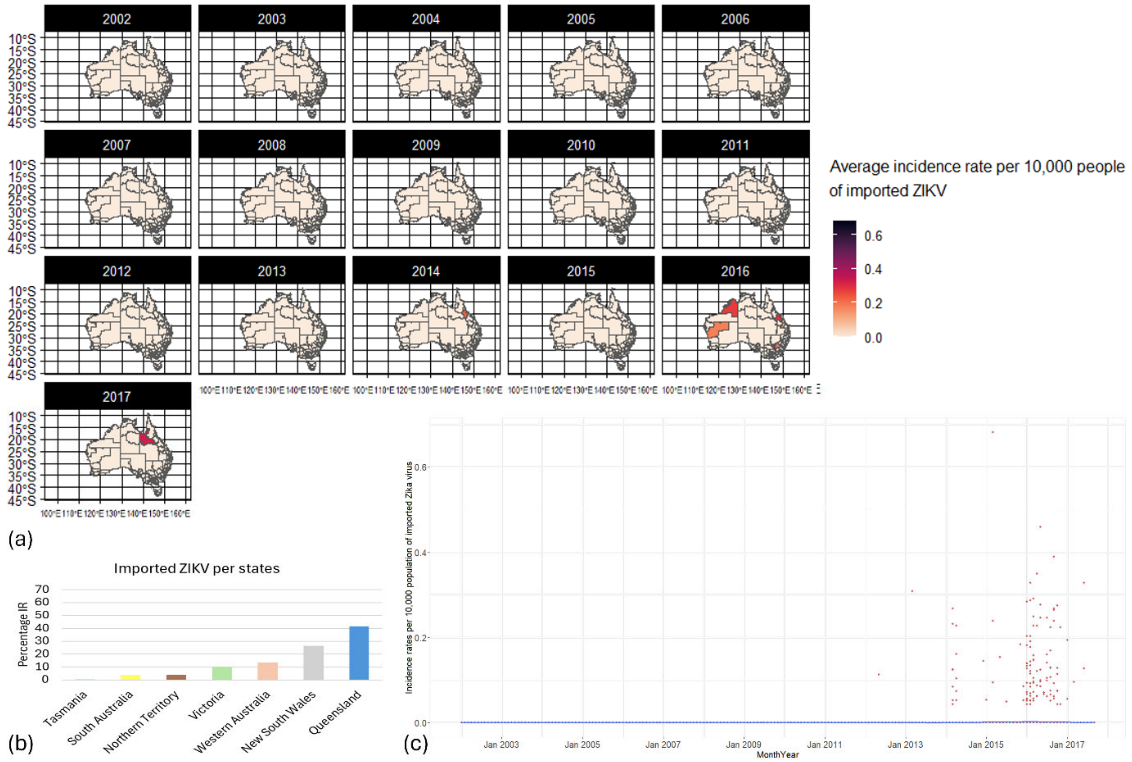

Imported Zika virus distribution from 2002 to 2017 in Australia. (**a**) Maps of the distribution of imported ZIKV cases per year through SA3s; (**b**) Top three states with the highest percentage of imported ZIKV local incidence rate; (**c**) Distribution of imported ZIKV incidence rate per 10,000 population through Australia; red dots: Incidence rate per 10,000 population per time and space; blue line: smoothed line to the plot. By default, geom_smooth() uses a Loess smoothing method for small datasets and a generalized additive model for larger datasets

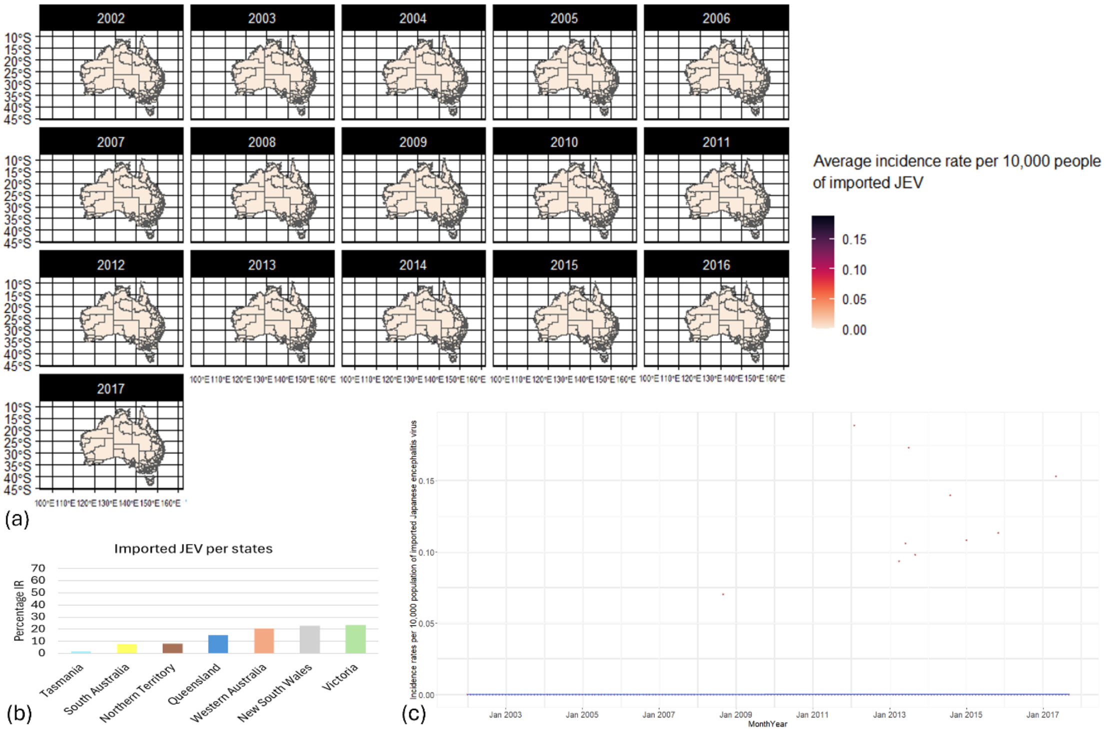

Figure 6. Imported Japanese Encephalitis virus distribution from 2002 to 2017 in Australia. (**a**) Maps of the distribution of imported JEV cases per year through SA3s; (**b**) Top three states with the highest percentage of imported JEV local incidence rate; (**c**) Distribution of imported JEV incidence rate per 10,000 population through Australia; red dots: Incidence rate per 10,000 population per time and space; blue line: smoothed line to the plot. By default, geom_smooth() uses a Loess smoothing method for small datasets and a generalized additive model for larger datasets

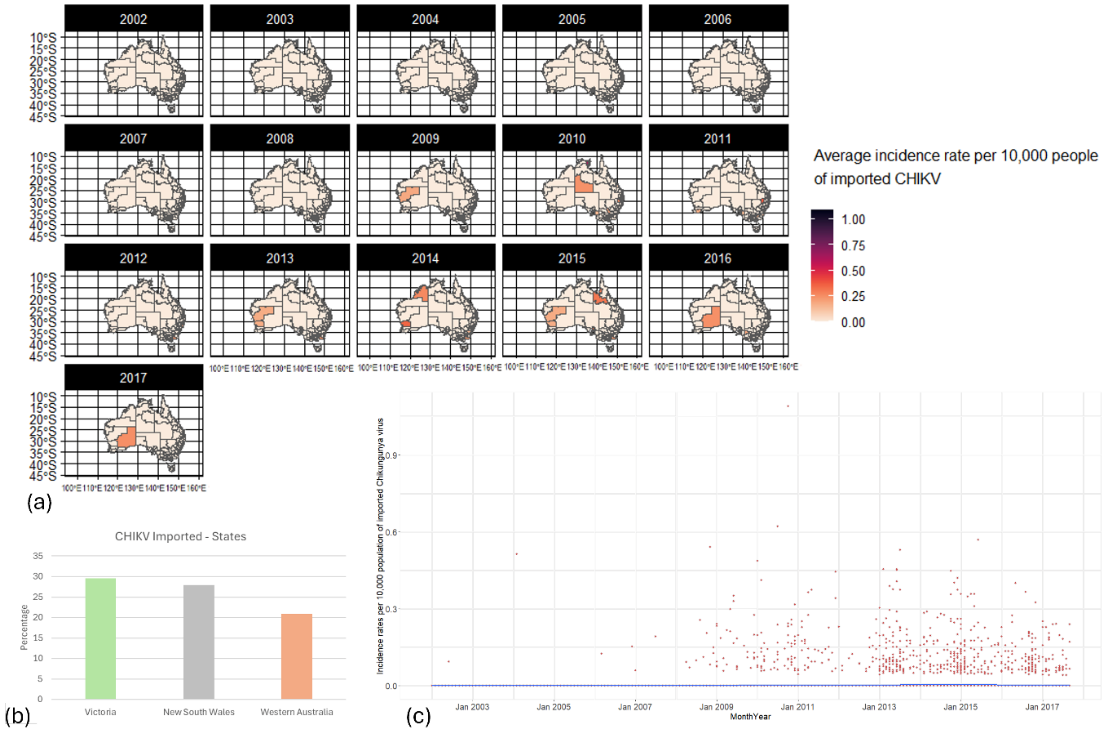

Figure 7. Imported chikungunya virus distribution from 2002 to 2017 in Australia. (**a**) Maps of the distribution of imported CHIKV cases per year through SA3s; (**b**) Top three states with the highest percentage of imported CHIKV local incidence rate; (**c**) Distribution of imported CHIKV incidence rate per 10,000 population through Australia; red dots: Incidence rate per 10,000 population per time and space; blue line: smoothed line to the plot. By default, geom_smooth() uses a Loess smoothing method for small datasets and a generalized additive model for larger datasets
