# Supplementary figures and images for: Arbovirus Transmission in Australia from 2002 to 2017
Source: Biology (Basel). 2024 Jul 15;13(7):524. doi: 10.3390/biology13070524 (PMC11273437; doi:10.3390/biology13070524)

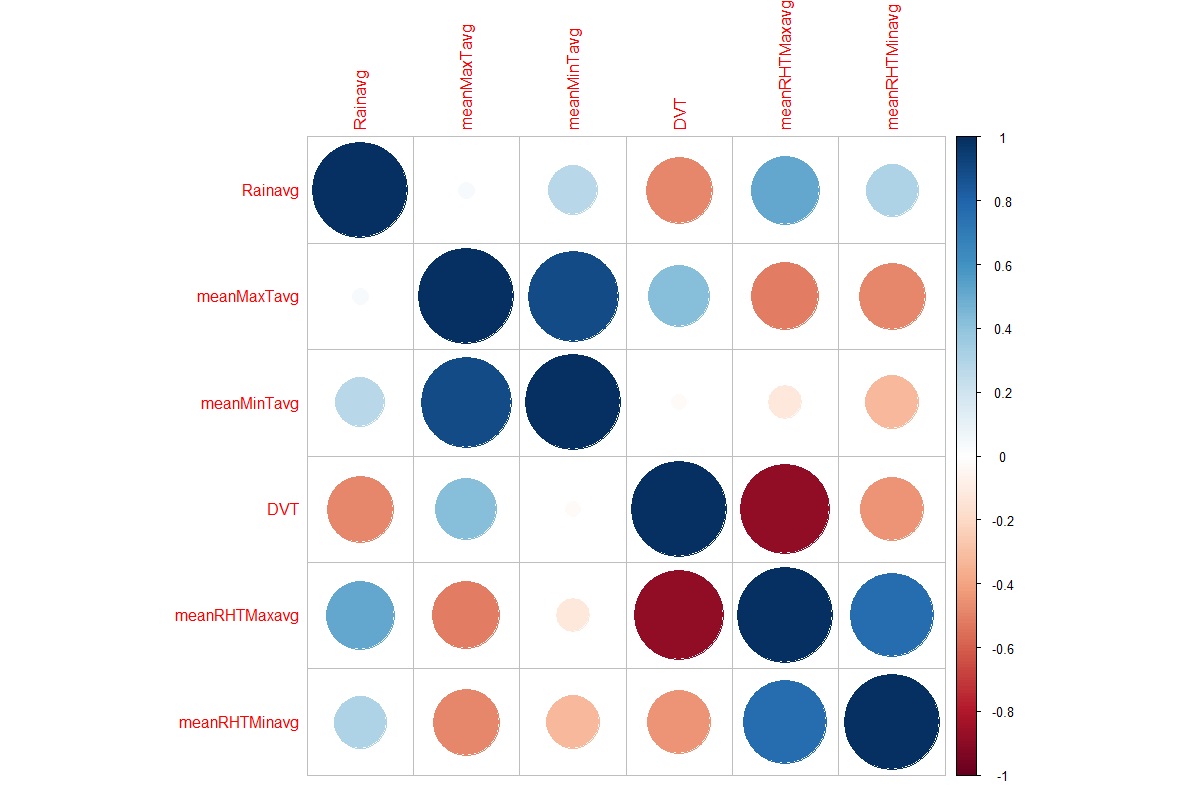

Supplement: Supplementary file 1 [file biology-13-00524-s001.zip › Revision Supl Mat/Figure S3 - correlation analysis.jpg]

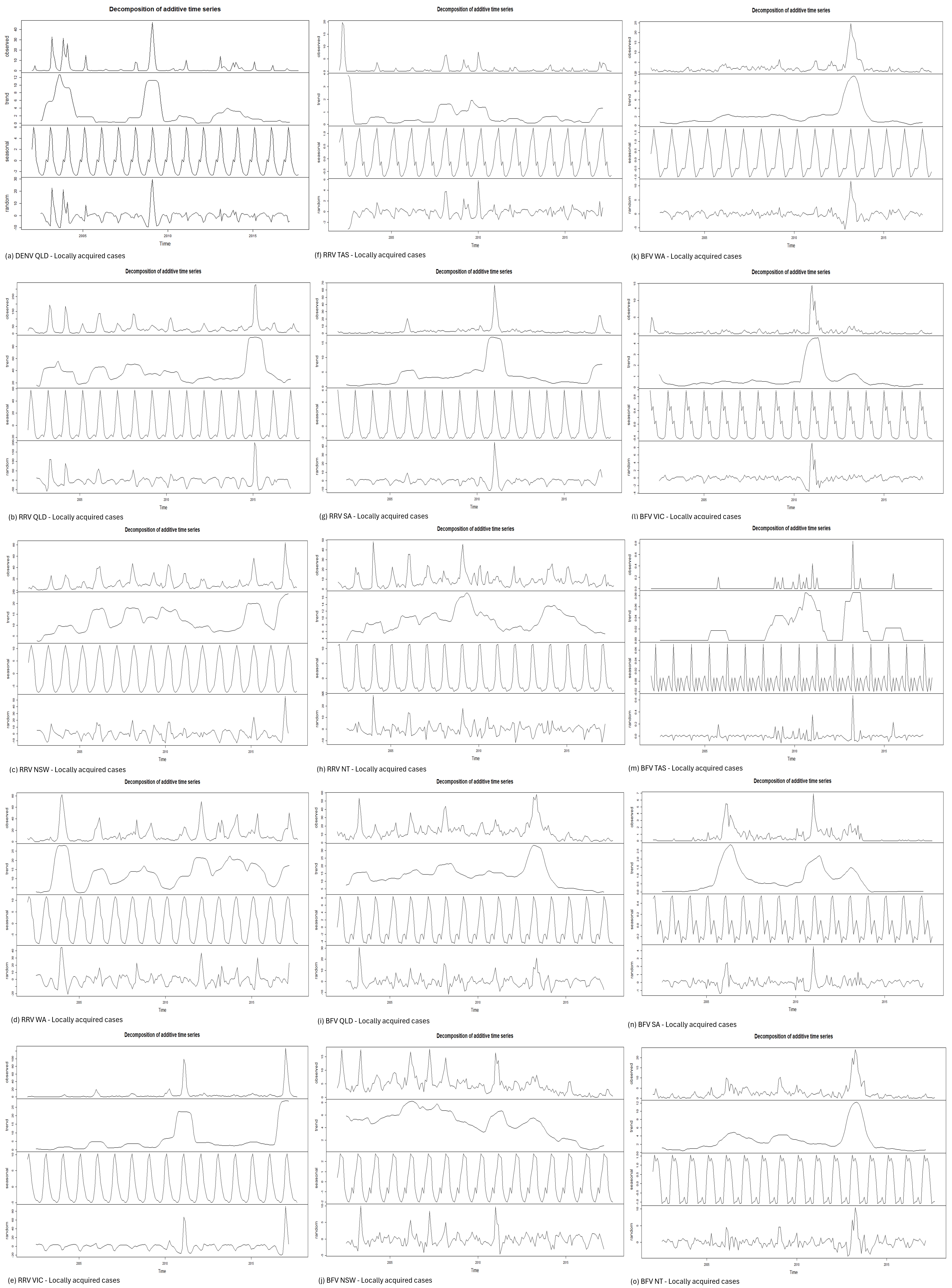

Supplement: Supplementary file 1 [file biology-13-00524-s001.zip › Revision Supl Mat/FigureS2 seasonality decomposition.jpg]
